# Comparison of infliximab with adalimumab for the treatment of non-infectious uveitis: a systematic review and meta-analysis

**DOI:** 10.1186/s12886-023-02987-1

**Published:** 2023-05-29

**Authors:** Weishai Liu, Dan Bai, Lieling Kou

**Affiliations:** 1 Department of Ophthalmology, Ankang Hospital of Traditional Chinese Medicine, No. 47 Bashan East Rd., Hanbin Dist, Ankang City, 725000 China; 2 Wenzhou Medical University, Wenzhou, China

**Keywords:** Infliximab, Adalimumab, Non-infectious uveitis, Efficacy, Safety

## Abstract

**Purpose:**

To compare the efficacy and safety of infliximab with that of adalimumab in the treatment of non-infectious uveitis (NIU).

**Methods:**

We searched for relevant studies in the PubMed, Embase, ClinicalTrials.gov, Cochrane Library databases, Grey Matters, Grey Literature Report, OpenGrey, China National Knowledge Infrastructure (CNKI), and Wan Fang databases up to September 2022. The incidences of complete remission of inflammation, response to therapy, adverse events and corticosteroid-sparing effect were evaluated.

**Results:**

Eleven clinical trials covering 1459 NIU patients were included. Complete remission of inflammation after therapy was achieved in 161 (37.5%) patients in the infliximab group and 151 (39.6%) patients in the adalimumab group. These two groups were not significantly different (*P* = 0.37). Four studies reported response to anti-TNF therapy involving 449 patients, of whom 241/272 (88.6%) treated with infliximab and 153/177 (86.4%) treated with adalimumab achieved partial or complete remission of inflammation. No significant difference was observed between the two cohorts in terms of response to therapy (P = 0.86). There was no significant difference between infliximab and adalimumab with regard to corticosteroid-sparing effect (P = 0.58). The pooled effect size (P = 0.001) showed a statistically significant difference, with the incidence of adverse events being 17.91% for infliximab and 12.12% for adalimumab.

**Conclusion:**

Our systematic review and meta-analysis of 11 studies suggests that infliximab and adalimumab have similar therapeutic efficacy and corticosteroid-sparing effect in patients with NIU. However, adalimumab has a marginal advantage over infliximab in terms of adverse events. Large-scale RCTs with a longer follow-up are required to further evaluate these two anti-TNF-α agents in patients with NIU.

## Introduction

Uveitis is a heterogeneous collection of intraocular inflammatory diseases of the uveal tract [1]. The incidence is estimated to be 17–52/100,000 person-years [2], and 22% of patients with uveitis are at risk of going blind at the same time [3]. Non-infectious uveitis (NIU) is relatively more common than infectious uveitis in both adults [4] and children [5]. In a recently published article, NIU contributed to 65.7% of all 1199 patients with uveitis, and another national registry report [5] from Turkey demonstrated that 333 of 442 (75.3%) children with uveitis were NIU. The etiologies of NIU vary widely, such as Behçet’s syndrome, juvenile idiopathic arthritis (JIA), idiopathic uveitis, Vogt-Koyanagi-Harada (VKH) syndrome. In general, the NIU is considered to be a group of autoimmune-mediated disorders [6].

Corticosteroids, which remain the mainstay of current therapy for NIU, are effective in eliminating inflammation in partial NIU patients, but not so effective in patients with severe and refractory uveitis [7]. Even in patients under control with daily doses of corticosteroids, long-term side effects are a major concern. Therefore, corticosteroid-sparing agents are urgently required.

Anti-tumor necrosis factor-alpha (anti-TNF-α) as a potent, multifunctional, monoclonal antibody not only plays a crucial role in exerting the homeostatic functions of the immune system, but also has shown fairly excellent anti-inflammatory efficacy to manage refractory NIU [8]. What’s more, it has corticosteroid-sparing effect [9]. Infliximab (Remicade) and adalimumab (Humira) are two primary anti-TNF-α biologics, and both are full-length bivalent IgG monoclonal antibodies. What distinguishes infliximab from adalimumab is that infliximab is a chimeric protein of 75% human-derived and 25% mouse-derived amino acids whereas adalimumab is a fully human-derived monoclonal antibody agent [10]. Both two agents, clinically adopted as mediators of inflammation, has successfully mitigated NIU in selected patients, but Adalimumab is the only one that has completed phase III studies and has been approved for the treatment of NIU by several countries including China, the United States, Japan and European countries [11].

There is little large-scale randomized-control, double-blind trials or meta-analyses to compare Infliximab with Adalimumab for the efficacy and safety in NIU treatment, though a large number of literatures have been published in this aspect. Hence, we herein performed a systematic review and meta-analysis in order to get a higher-level evidence to evaluate the efficacy and safety of these two anti-TNF-α agents for the treatment of NIU.

## Methods

This systematic review and meta-analysis was carried out in accordance with the preferred reporting items for systematic review and meta-analysis (PRISMA) statements and the protocol adhered to the PRISMA protocol guidelines.

### Search strategy

We conducted a systematic search in the PubMed, Embase, ClinicalTrials.gov, Cochrane Library databases, with language restricted to English, China National Knowledge Infrastructure (CNKI), and Wan Fang databases, with language restricted to Chinese, from database inception to September 15, 2022 to identify potential papers that compared infliximab with adalimumab treatment for NIU. To ensure inclusion of all potential studies, the grey literature was also searched in three databases including Grey Matters (https://www.cadth.ca/resources/finding-evidence/grey-matters), Grey Literature Report (http://www.greylit.org/home), and OpenGrey (http://www.opengrey.eu). Appropriate methods were used, which included the application of Medical Subject Headings (MeSH) and free words related to NIU, infliximab and adalimumab, including “non-infectious uveitis”, “NIU”, “Adalimumab”, “Humira”, “Infliximab”, “Remicade”, “anti-tumor necrosis factor-alpha”, “anti-TNF-α”. The search was limited to the title, abstract and keyword fields to filter out irrelevant studies.

### Inclusion criteria and exclusion criteria

Inclusion criteria were as follows: (1) randomized controlled trials or retrospective studies comparing infliximab with adalimumab treatment in patients of any ethnicity, gender, or age with a diagnosis of NIU; (2) studies containing infliximab and adalimumab as the same cohort compared with other immunosuppressants, but the data of infliximab and adalimumab could be extracted separately; (3) studies that comprise one or more than one type of NIU, regardless of chronic, severe or refractory NIU; (4) the mean follow-up time was more than 6 months; 4) studies that have at least 10 patients with NIU in each group to avoid bias.

Exclusion criteria were defined as follows: (1) duplicate reports of the same study; (2) studies in which baseline information or outcomes were not clearly stated; (3) inability to extract the data from the paper; (4) reviews and meta-analyses.

### Outcome measures

The outcome measures of efficacy included the proportion of NIU patients with remission of inflammation and assessment of corticosteroid-sparing effect. The outcome measure of safety was assessed by the incidence of adverse events. Adverse events included, but not limited to: (1) allergic reactions of injection site; (2) new-onset or reactivated infection; (3) gastrointestinal discomfort; (4) adverse event-related death.

### Study selection and data extraction

Two reviewers (W.S.L. and D.B.) independently screened the titles and abstracts of all searched items and all potentially relevant articles with full text were retrieved for further assessment in accordance with the predetermined inclusion and exclusion criteria. Extraction table was used to collect extracted data from the included studies which were sorted by publication date. Disagreements between the two reviewers were resolved through discussion. If not, consensus was made through consultation with a third reviewer. The following data were extracted: (1) name of the first author; (2) date of publication; (3) design of study; (4) country of study; (5) sample size; (6) age of individuals; (7) length of follow-up; (8) type of anti-TNF-α agents; (9) type of NIU; (10) number of patients achieving complete remission of inflammation; 11) number of patients achieving corticosteroid reduction; 12) number of patients with adverse events.

### Risk of bias and data analysis

The quality and risk of bias of each study were evaluated using the Methods Guide for Systematic Reviews of Medical Tests from the Agency for Healthcare Research and Quality (AHRQ) [12]. The total number of included patients who experienced the outcome was counted, and all dichotomous variables, reported as rates, including complete remission of inflammation and adverse events, were preferentially employed in this meta-analysis as odds ratio by Mantel-Haenszel with fixed-effects model along with 95% confidence intervals (95% CIs). For continuous variables including corticosteroid-sparing effect, we retrieved the mean and standard deviation (SD) of corticosteroid-sparing effect by formulae which uses the sample size, median, interquartile range [13]. The pooled mean and SD were estimated by heterogeneity, which determines a random-effects or a fixed-effects model to be adopted according to the results of *I* ^2^ test and chi-square-based Q test. When *I* ^2^ > 50% and *P* < 0.05, a random-effects model was used; otherwise, a fixed-effects model was adopted. Sensitivity analysis was conducted by omission of specific studies. All the statistical analyses were conducted with the aid of RevMan 5.4 software (Cochrane Library Software, Oxford, UK). Potential publication bias was assessed using funnel plots and the Egger’s test which was performed with the support of STATA 14.0 software (StataCorp, College Station, TX, USA).

## Results

### Study selection

**Fig. 1:**
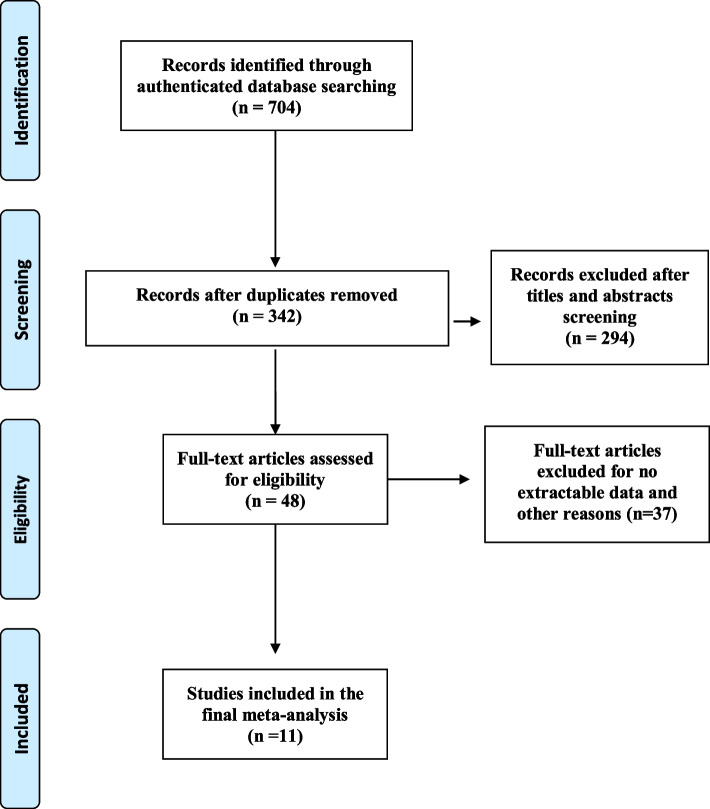
Flowchart of selection procedure

A total of 704 references were retrieved from all of the aforementioned databases. After screening out 362 duplicate records, 342 items were left for further evaluation by browsing abstracts and titles. Subsequently, 48 potentially relevant articles were carefully reviewed by reading the full text, and 37 papers were scrapped for a variety of reasons, including absence of outcomes of interest, non-extractable results, sample size less than 10, and papers with the possibility of sharing the same cohort of already-included studies. Finally, 11 studies were selected for meta-analysis [14–24]. The flowchart of the selection process and the reasons for exclusion are demonstrated in Fig. 1.

### Characteristics of included studies

The characteristics of the included studies are summarized in Table 1. Of the 11 studies published between 2011 and 2022, 9 were of retrospective design and the other 2 were prospective studies, involving 1459 patients (788 females (54%) and 671males (46%)). 777 NIU patients were treated with infliximab and 682 treated with adalimumab. All studies were conducted in European countries, except one in Turkey [22] and another one in Japan [21]. One study [21] did not disclose the mean follow-up time, the others ranged from 0.5 to 3 years. The mean age was reported by total population or by treatment type and ranged from 8.8 to 57.6 years. The top 2 causes of NIU are extracted and listed in the column of “Type of NIU”. Infliximab was administered intravenously with a loading dose of 3-5 mg/kg at 0, 2, 6 weeks, and continued every 4 to 8 weeks. Adalimumab was administered subcutaneously at a dose of 40 mg every 2 weeks with or without a loading dose of 80 mg.

**Table 1:** Characteristics of included studies

Author (year)	Location	Design	Number of Patients		Type of NIU	Follow-up (year)	Age (Mean±SD or Median [IQR]) (Infliximab / Adalimumab)	Male/Female
			Infliximab	Adalimumab				
Simonini, et al. (2011) [14]	Italy	Prospective, comparative case series study	17	16	JIA; Idiopathic; Other	3	9.17 [IQR NA]	11/22
Zannin, et al. (2013) [15]	Italy	Prospective study	48	43	JIA	1	10.5 ± 4.3 / 8.8 ± 4.4	20/71
Rio, et al. (2014)	Spain	Interventional case series multicenter study	77	47	Behçet’s disease	1	38.6 ± 10.4	68/56
Vallet, et al. (2015) [17]	France	Retrospective multicenter study	77	47	Behçet’s disease	3	33.5 [IQR 28,40]	60/64
Vallet, et al. (2016) [18]	France	Multicenter retrospective observational study	98	62	Behçet's disease; JIA; Other	1	31 [IQR 21-42]	62/98
Fabiani, et al. (2018) [19]	Italy	Retrospective observational study	41	66	Behçet's disease; Idiopathic; Other	1	42.15 ± 12.14 / 39.52 ± 12.08	61/46
Mateo, et al. (2019) [20]	Spain	Multicenter observational study	103	74	Behçet’s disease	1	40.4±10.1	94/83
Kunimi, et al. (2020) [21]	Japan	Single-center retrospective study	68	63	Behçet's disease; Sarcoidosis; Other	NA	39.5 ± 14.6 / 57.6 ± 15.2	78/53
Gunduz, et al. (2021) [22]	Turkey	Single-center retrospective cross-sectional study	16	17	JIA; Idiopathic; Other	2	15.2 ± 4.7 / 12.5 ± 4.2	5/28
Leclercq, et al. (2021)	France	Multicenter retrospective observational study	69	80	Idiopathic; Behçet's disease; Other	0.5	40 [IQR 28,58]	63/86
Maalouf, et al. (2022) [24]	France	Multicenter retrospective observational study	163	167	Idiopathic; Behçet's disease; Other	0.5	36 [IQR 27,54]	149/181

*NIU *Non-infectious uveitis, *SD* Standard deviation, *IQR* Interquartile range, *NA* Not available, *JIA* Juvenile idiopathic arthritis

### Meta-analysis and small-study effects

#### Complete remission of inflammation

**Fig. 2:**
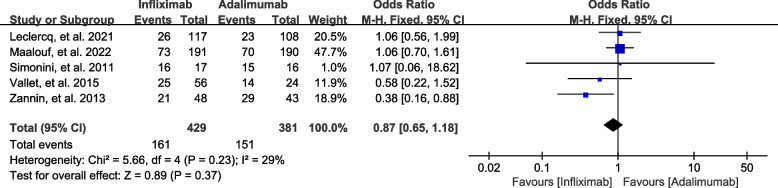
Forest plot of complete remission of inflammation in NIU patients treated with infliximab vs. adalimumab

Complete remission of inflammation was defined as the absence of active uveitis (grade 0 for vitreous haze and anterior chamber cells) for more than 6 months. Complete remission of inflammation after infliximab therapy at 1 year or at the last evaluation was achieved in 161 (37.5%) patients from 5 studies with 429 patients, and 151 of 381 (39.6%) patients achieved complete remission of inflammation in the pooled cohort of adalimumab. As shown in Fig. 2, there was little heterogeneity (*I* ^2^ = 29%, *P* = 0.23). The pooled complete remission of inflammation between these two groups was not significantly different (*P* = 0.37).

#### Response to therapy

**Fig. 3:**
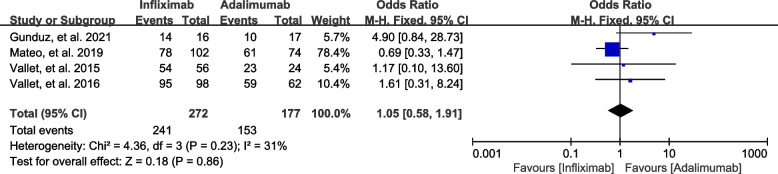
Forest plot of response to therapy in NIU patients treated with infliximab vs. adalimumab

The term “response to therapy” encompasses both complete and partial remission of inflammation in anti-TNF therapy. Two studies were excluded from the efficacy analysis of response to therapy, as Kunimi, et al. [21] assessed efficacy by mean change in visual acuity and Fabiani, et al. [19] assessed efficacy by mean change in best corrected visual acuity, rather than response rate. Additionally, Rio, et al.’ study [16] was dropped because they didn’t report the response to infliximab and adalimumab therapy separately. As a result, four studies reported response to anti-TNF therapy involving 449 patients, of whom 241/272 (88.6%) treated with infliximab and 153/177 (86.4%) treated with adalimumab achieved partial or complete remission of inflammation. No significant difference was observed between the two cohorts in terms of response to therapy (P = 0.86). Figure 3 illustrates that there was no significant heterogeneity (I2 = 31%, P = 0.23).

#### Corticosteroid-sparing effect

**Fig. 4:**
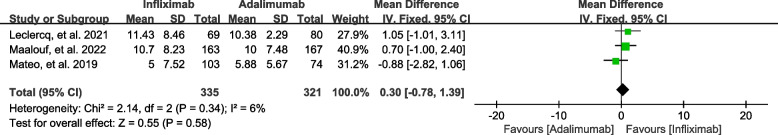
Forest plot of mean daily corticosteroid dose in NIU patients treated with infliximab vs. adalimumab at the end of the follow-up. (The studies by Leclercq, et al. and Maalouf, et al. were at six months; The study by Mateo, et al. was at one year.)

Corticosteroid-sparing effect was defined as a reduction in the daily corticosteroid dose required from the start of treatment with biological agents to the last assessment in the treatment of NIU. A total of 656 patients from 3 studies were adopted to compare infliximab with adalimumab in terms of corticosteroid- sparing effect. All the available data extracted from the original articles were converted and expressed as mean ± standard deviation (SD) using formulas [13, 25]. The mean daily corticosteroid dose at the end of the follow-up was pooled. (The studies by Leclercq, et al. and Maalouf, et al. were at six months; the study by Mateo, et al. was at one year.) There were no significant differences between infliximab and adalimumab as far as corticosteroid-sparing effect (*P* = 0.58) and heterogeneity (*I* ^2^ = 6%, *P* = 0.34) are concerned (Fig. 4).

#### Adverse events

**Fig. 5:**
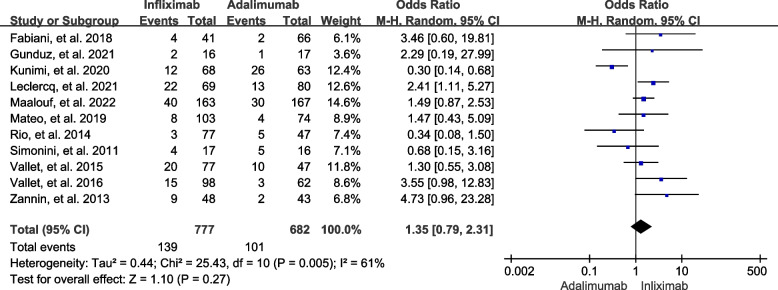
Forest plot of adverse events in NIU patients treated with infliximab vs. adalimumab (with Kunimi, et al. study)

**Fig. 6:**
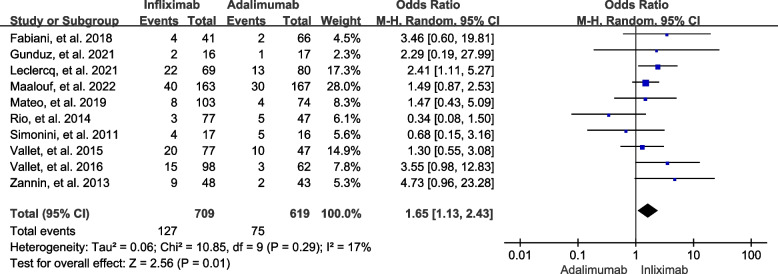
Forest plot of adverse events in NIU patients treated with infliximab vs. adalimumab (without Kunimi, et al. study)

All 11 included studies consisting of 1459 patients were systematically reviewed for adverse events. Of these, 240 (16.45%) cases had adverse events that were adjudicated by the reviewers or the original authors. The meta-analysis data showed that there was a significant heterogeneity (*I* ^2^ = 61%, *P* = 0.005), so a random-effects model was chosen. There was no statistically significant difference in the incidence of adverse events between the two groups (OR = 1.35, 95% CI: 0.79 to 2.31, *P* = 0.27) (Fig. 5). We then performed a sensitivity analysis for adverse events. The heterogeneity (*I* ^2^ = 17%, *P* = 0.29) and pooled effect size (*P* = 0.001) showed statistically significant changes, with the incidence of adverse events being 17.91% for infliximab and 12.12% for adalimumab when we omitted the data from Kunimi, et al. [21] (Fig. 6), but not changing much when we omitted others.

#### Small-study effects

**Fig. 7:**
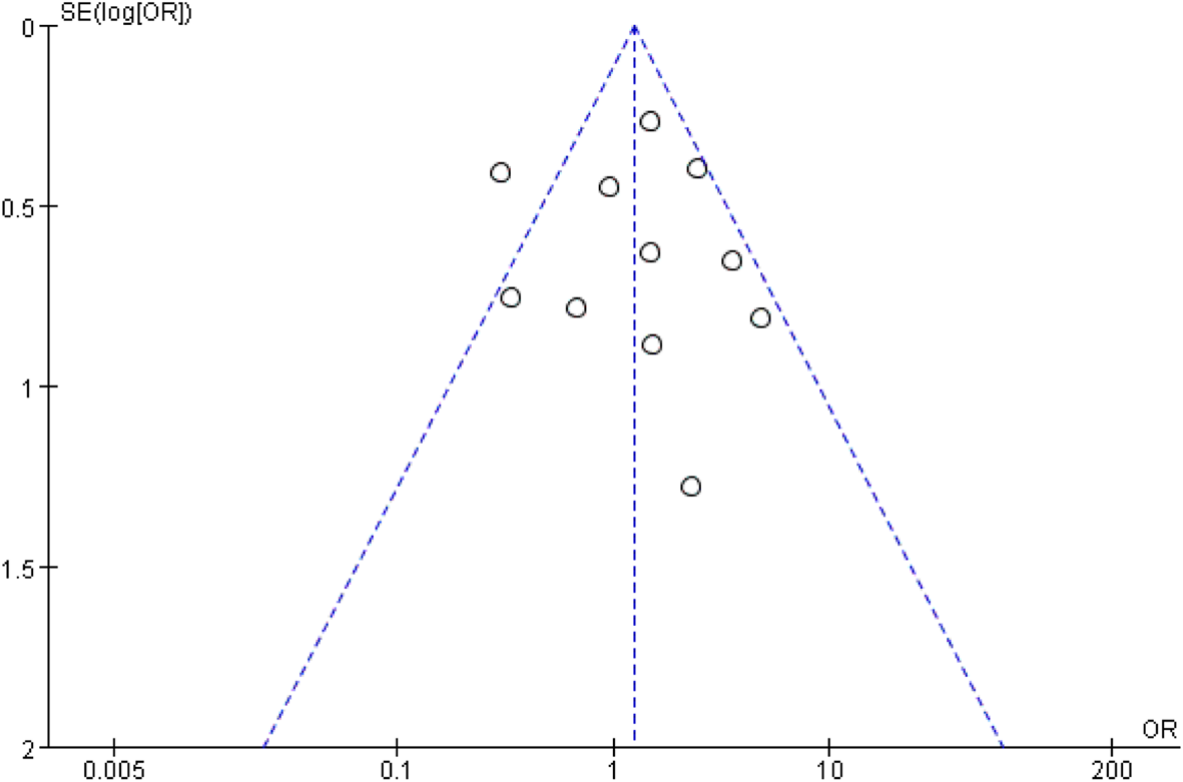
Funnel plot of adverse events

We assessed the small-study effects using a funnel plot of adverse events. According to the basically symmetrical funnel plot, no significant small-study effects were found which was corroborated by the results of Egger’s test (*P* = 0.846) (Fig. 7).

## Discussion

TNF-α is a pro-inflammatory cytokine produced by many cell types including almost all cells of the immune system and can bind to pigment epithelial cells of the retina, ciliary body and iris, leading to breakdown of the blood-ocular barrier. Under physiological conditions, serum levels of TNF-α are undetectable, but levels increase significantly in the aqueous humor and serum following an inflammatory stimulus such as NIU. The anti-TNF-α biologics infliximab and adalimumab can substantially neutralize TNF-α and markedly suppress ocular inflammation. To date, several meta-analyses have been published focusing mainly on the overall efficacy and safety of anti- TNF-α agents, but none of them compared the efficacy and safety between these agents. In the current meta-analysis, we collected all the available evidence on the use of infliximab and adalimumab in the same trial to evaluate their efficacy and safety in the treatment of NIU.

The pooled incidence rates of complete remission of inflammation in the infliximab and adalimumab groups were 37.5% and 39.6%, respectively, and of response to therapy (complete and partial remission of inflammation) were 88.6% and 86.4%, respectively. These data indicate that both infliximab and adalimumab can substantially suppress the inflammation but completely reduce inflammation only in partial patients (less than 50% of NIU patients). In addition, they were shown to have similar benefits for responders in terms of complete remission of inflammation (*P* = 0.37) and response to therapy (*P* = 0.86). The response to therapy appears to be higher than that in other meta-analyses where the proportions of inflammation remission were 68% with anti-TNF-α therapy in the review published by Hu, et al. [26] and 79% in another study [27] focusing on adalimumab. One possible reason is that we extracted the complete remission from response to therapy. If a study with only complete remission is pooled with other studies with response to therapy, there will be a relatively lower rate of inflammatory remission. Another reason may be that we included children with NIU in this study. Children with NIU have a lower prevalence of severe complications [28], and have a higher response rate which has been supported by two reviews [29, 30]. One study [29] summarized the literature spanned from Jan 2000 to Oct 2012 with the proportion of responding children being 87% for adalimumab and with no significant difference from infliximab (72%), while another [30] focusing on NIU children collected the literature spanned from Nov 2012 to Jan 2020 with the proportion of responders being 86% for adalimumab and with a significant difference from infliximab (68%). However, there was no such a significant difference between infliximab and adalimumab in the current study, which may be due to the inclusion of both adults and children with NIU.

We also evaluated the corticosteroid-sparing effect according to the currently available studies. Most studies reported the daily corticosteroid dosage with median and interquartile range, so we converted the data to mean ± SD according to the aforementioned literature [13, 25]. The mean dosages at the last follow-up were recorded and calculated. The pooled meta-analysis demonstrated that infliximab and adalimumab have similar corticosteroid-sparing effect (*P* = 0.58). Before drawing a firm conclusion from this result, one caveat must be noted. Although the formulas are scientific, the process of conversion inevitably introduces errors. We did not analyze the mean daily dose of corticosteroid reduction from the initial visit to the last follow-up. Because multiple conversions would amplify the errors and lead to no meaningful results. We also did not categorize the corticosteroid-sparing effect into corticosteroid-suspended and corticosteroid-tapered subtypes because of the limited number of studies reporting details.

The safety of anti-TNF-a is an aspect that cannot be neglected. We reviewed all the 11 included studies and analyzed the results for adverse events. The pooled results showed that 16.45% of the cases experienced at least one adverse event. A sensitivity analysis was performed because a significant heterogeneity (*I* ^2^ = 61%, *P* = 0.005) was identified during the process of analysis. Ultimately, we found that the study published by Kunimi, et al. [21] contributed to the heterogeneity. After careful examination of this study, the possible reason is that they included more infusion/injection reactions in the adverse events. 11 of 12 patients (91.7%) in the infliximab group and 22 of 26 patients (84.6%) in the adalimumab group had infusion/injection reactions. After exclusion of this study, adalimumab seems to be in advantage over infliximab in terms of adverse events (*P* = 0.001). The advantage appears to be marginal. Because there was no significant difference (*P* = 0.27) with all these studies using a random-effects model. The results should therefore be interpreted with caution.

With regard to individuals, particular attention should be paid to elderly patients. As anti-TNF-α agents have the potential to increase the risk of serious infections such as bacteremia, pneumonia, tuberculosis [31–33]. The use of anti-TNF-α agents is not recommended by the New York Heart Association (NYHA) for patients with class III and IV heart failure [34]. In addition, an increased incidence of melanoma and lymphoma has been observed in patients taking anti-TNF-α agents [16, 17, 20, 24]. These rare but fatal adverse events call for vigilance during follow-up period. Regarding treatment costs, the annual cost of infliximab (15,799 Euro) is slightly higher than that of adalimumab (12,731 Euro) in the following strategy: Infliximab was administered intravenously with a loading dose of 5 mg/kg at 0, 2, 6 weeks, and continued every 8 weeks. Adalimumab was administered subcutaneously at a dose of 40 mg every 2 weeks [18].

### Limitations

Most studies with 1 year of follow-up are not sufficient to comprehensively and precisely evaluate the efficacy and safety of infliximab and adalimumab. This is because infliximab has been observed to be effective in the short-term, and its efficacy seems to wane over time in children with NIU [35, 36]. As a consequence, higher doses of infliximab have been used to treat NIU in long-term follow-up [37, 38]. None of these included studies is a randomized controlled trial (RCT), which is another limitation of this meta-analysis. Hence, additional RCTs with a longer follow-up are warranted.

## Conclusion

Our systematic review and meta-analysis of 11 studies suggests that infliximab and adalimumab have similar therapeutic efficacy and corticosteroid-sparing effect in patients with NIU. However, adalimumab has a marginal advantage over infliximab in terms of adverse events. Large-scale RCTs with a longer follow-up are required to further evaluate these two anti-TNF-α agents in patients with NIU.

## Data Availability

The datasets used and/or analyzed during the current study available from the corresponding author on reasonable request.
